# Laparoscopic sacral hysteropexy for pelvic organ prolapse in a patient affected by marfan syndrome: a case report

**DOI:** 10.52054/FVVO.13.4.043

**Published:** 2021-12-30

**Authors:** G Campagna, L Vacca, D Caramazza, G Panico, S Mastrovito, G Scambia, A Ercoli

**Affiliations:** Fondazione Policlinico Universitario A. Gemelli IRCCS, UOC Uroginecologia e Chirurgia Ricostruttiva del Pavimento Pelvico, Dipartimento di Scienze della Salute della Donna e del Bambino e di Sanità Pubblica, Roma, Italia, 00168; Fondazione Policlinico Universitario A. Gemelli IRCCS, UOC Uroginecologia e Chirurgia Ricostruttiva del Pavimento Pelvico, Dipartimento di Scienze della Salute della Donna e del Bambino e di Sanità Pubblica, Roma, Italia,00168; Fondazione Policlinico Universitario A. Gemelli IRCCS, UOC Uroginecologia e Chirurgia Ricostruttiva del Pavimento Pelvico, Dipartimento di Scienze della Salute della Donna e del Bambino e di Sanità Pubblica, Roma, Italia,00168; PID Ginecologia Oncologica e Chirurgia Ginecologica Miniinvasiva, Università degli studi di Messina, Policlinico G.Martino, Messina, Italia ,98124

**Keywords:** pelvic organ prolapse, Marfan syndrome, hysteropexy, laparoscopy

## Abstract

**Background:**

Marfan Syndrome (MS) is a dominantly inherited connective tissue disorder with consequences on the strength and resilience of connective tissues that may predispose to Pelvic Organ Prolapse (POP). Literature lacks studies investigating POP surgery in patients affected by MS that might help surgical management decisions.

**Objective:**

The objective of this paper is to describe the surgical procedure of laparoscopic sacral hysteropexy (LSHP) in a 37 years old woman affected by MS with symptomatic POP.

**Materials and Methods and main outcome measures:**

We performed a nerve-sparing laparoscopic sacral hysteropexy without complications and looked for anatomical and subjective outcomes. The patient completed The Female Sexual Distress Scale (FSDS), Pelvic Floor Disability Index (PFDI-20), and Wexner questionnaires preoperatively and postoperatively.

**Results:**

The patient stated a complete resolution of all POP related symptoms and there was a total correction of the descensus. Furthermore, no perioperative and postoperative complications were noted.

**Conclusions:**

LSHP could be an effective and safe procedure for the treatment of POP in women affected by MS and this case report is the first to describe a reconstructive procedure in this category of patients.

**What is new?:**

The literature lacks studies investigating POP surgery in women with MS, that might help surgeons, thus we present this case to describe surgical and functional outcomes in this patient category, underlying the higher risk of complications and relapses related to the weakness of connective tissue. This case report may represent the basis of future studies to confirm the safety, efficacy and feasibility of LSHP and sacral colpopexy in patients with MS.

## Introduction

Pelvic organ prolapse (POP) has a complex and multifactorial aetiology. Although published studies suggest that several diseases contribute to the loss of structural support to pelvic organs, mechanisms underlying it are still poorly understood (Vergeldt et al., 2015). According to published data, defects in the extracellular matrix (ECM) or fibrous connective tissue are involved in the genesis of POP due to the consequent decreased tissue strength and altered repair (Eser et al., 2015). Particularly extracellular matrix (ECM) which is largely built of elastic fibers and microfibril proteins such as fibrillins. Fibrillins represent a small family of proteins that are integral constituents of the non-collagenous microfibrils which are distributed in the extracellular matrix, contributing to the integrity and function of all connective tissues (Cecati et al., 2018). Microfibrillar bundles provide the external coating to elastin in elastic fibers, serving as scaffolds for elastin deposition and as an anchoring function in non-elastic tissues (Ramirez et al., 2004). Fibrillin production may be altered by genetic defects generating complex disorders that influence tissue growth and homeostasis (Sandberg et al., 1971).

Marfan syndrome (MS) is a relatively common (3:5000) dominantly inherited disorder of connective tissue, caused by mutation of FBN1, encoding the gene for fibrillin-1, with variable clinical features in the musculoskeletal, cardiovascular and ocular systems. A cardinal and potentially life-threatening aspect of MS is aortic root aneurysm with subsequent dissection and rupture. Other important clinical features include mitral valve prolapse, pneumothorax, dural ectasia, and myopia (Judge and Dietz, 2005). The consequences of this mutation on the strength and resilience of the connective tissue may predispose to POP as suggested by its higher prevalence in women with MS, implying a direct correlation between pelvic floor strength and levels of Fibrillin-1 expression (Eser et al., 2015). However, literature lacks studies investigating POP surgery in patients affected by MS, that might help surgical management decisions.

For these reasons, we present the first case of a young woman affected by MS undergoing laparoscopic sacral hysteropexy (LSHP) for POP to describe its surgical and functional outcomes in this category of patients.

## Case presentation

A 37-year-old nulliparous woman with MS was referred to our Urogynecological department of Fondazione Policlinico Universitario A. Gemelli IRCCS for POP and underwent nerve-sparing LSHP.

She was Caucasian with a body mass index (BMI) of 16.5 Kg/m^2^ and was affected by symptomatic POP (POP-Q: Aa: 3, Ba: 3, C: +5, Ap: 2; Bp:2, Pb: 3, Gh: 4, D: 4, TVL: 10) and urethral hypermobility. She complained of frequency, nocturia, sense of incomplete bladder emptying, hesitancy, dysuria, vaginal bulging, dyspareunia, constipation, obstructed defecation syndrome, without fecal or urinary incontinence. Previous surgical procedures included only tonsillectomy. She was affected by aortic root dilatation and mild mitral valve prolapse related to her pathology.

At the urodynamic testing the filling phase was normal while the emptying phase was obtained with the help of the abdominal muscles, demonstrating a bladder neck obstruction with a reduced maximum flow rate (Q max 10 ml/s) and a post voided residual (PVR) of urine of 150 ml. The ultrasound evaluation revealed a mild left hydronephrosis, with normal uterus, ovaries, bladder and kidneys and the smear test was normal. Since the patient was complaining obstructed defecation syndrome, we required a defecography that excluded the presence of internal rectal prolapse or intussusception. Patients before undergoing the described procedure, received accurate surgical counseling, where they are given information on different surgical approaches, and are advised about the risks of mesh positioning. This patient signed an informed consent allowing the use of personal data.

The Female Sexual Distress Scale (FSDS) (Derogatis et al. 2002), Pelvic Floor Disability Index (PFDI-20) (Barber et al., 2005), and Wexner (Rockwood et al., 2000) questionnaires were administered to the patient.

The surgical procedure was carried out as we previously described (Campagna et al., 2018; Panico et al., 2020), but since the uterus was preserved the two meshes were passed through a window created in the right broad ligament before the suspension, and was completed by a surgeon with high expertise in laparoscopic reconstructive surgery (more than 50 procedure per year). We used an open transumbilical laparoscopic access technique, with one transumbilical 10-mm port and three 5-mm ancillary ports to perform the surgery and 3D-high-definition 0° 10-mm scope for the intra-abdominal visualisation.

**Figure 1:**
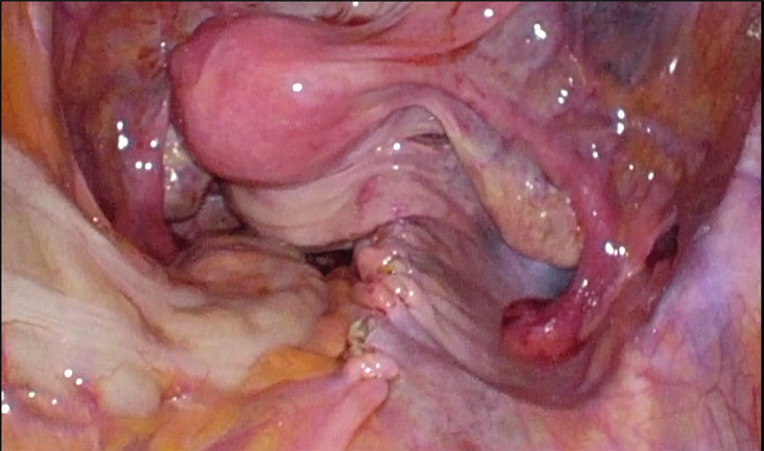
Appearance of pelvic organs at the completion of hysteropexy.

Firstly, we exposed the longitudinal vertebral ligament by opening the parietal peritoneum covering the sacral promontory. Peritoneal incision was prolonged along the right pelvic wall up to the uterine isthmus. The Douglas pouch was incised, and the rectovaginal space was fully dissected. At its caudal edge lateral to the rectum upward we identified the pelvic parietal fascia covering the levator ani muscle. An Y shaped polypropylene type 1 mesh (UpsylonTM Y-Mesh, Boston Scientific) was placed and fixed to the posterior vaginal wall by four 3–0 non-absorbable sutures (Ethibond, Ethicon Inc., Somerville, NJ, USA) to cover the entire dissection space without tension. Sutures were applied on the levator ani muscles and on the upper portions of the posterolateral vaginal walls. The vesico-uterine peritoneum was opened, and vesicovaginal space was dissected. The right broad ligament was fenestrated (unilateral fenestration) at the level of the cervico- uterine junction in an avascular space lateral to the uterine artery to thread the cephalad portion of the anterior mesh. The mesh was inserted on the dissection space and fixed to the anterior vaginal wall with 3–0 non absorbable sutures (Ethibond). Two to three 2– 0 non-absorbable sutures (Ethicon, Inc., Somerville, NJ). were placed on the anterior and posterior aspects of the cervix. The anterior mesh was threaded up toward the promontory from the vagina under visual control to lift the vagina. The mesh was fixed to the longitudinal vertebral ligament anterior to the L5–S1 intervertebral space with 1–0 non-absorbable suture (Ethicon, Inc., Somerville, NJ). The last step was the peritonealization of the mesh with a 2/0 barbed suture (Strata x spiral monocryl plus knotless tissue control device, Ethicon Inc. Somerville, NJ, USA). (Figure 1).

Total operating time (OT) was 130 min with an estimated blood loss of 50 ml. We didn’t register any perioperative complications. Time to discharge was 2 days. Post procedural ileus was 30 hours. At the discharge visit there was a total correction of the descensus (POP-Q: Aa: -3, Ba: -2, C: -8, Ap: -3; Bp:-3, Pb: 3, Gh: 3, D: 4 TVL: 10) (Muir, Stepp, and Barber 2003). The woman referred a complete resolution of all POP related symptoms.

At one year follow up the urogynecological examinations confirmed the postoperative anatomical outcome with apex well suspended and vaginal wall perfectly lifted. The patient completed the same preoperative questionnaires. Compared to the preoperative scores, PFDI-20 score decreased from 132.3 to 28.3, Wexner score from 10 to 4 and FSDS score from 38 to 18 and no dyspareunia was complained, supporting the improvement in POP related symptoms already referred during patient’s interview. Urodynamic testing showed a normal filling and voiding phase (Q max 25 ml/s) and the absence of PVR volume.

## Discussion

The role of Fibrillin-1 in pelvic floor support has already been suggested in literature (Eser et al., 2015). A decreased gene expression and weaker immunoreactivity for fibrillin-1 was found in women with Stress Urinary Incontinence. Loss of tissue elasticity might lead to increased urethra hypermobility and SUI (Söderberg et al. 2010). In another study published by Eser et al. (2015), no significant reduction in Fibrillin-1 expression was found in patients affected by POP, however fibrillin levels were found to be indicative of the risk of pelvic prolapse development when considered together with factors such as age and menopause stage (Eser et al., 2015).

From an in-depth research of the published literature on POP surgery in MS, inherited connective tissue disorders and POP surgery, a lack of specific data available on the relationship between this genetic disease and POP and its management has emerged.

Carley and Schaffer (200) evaluated the prevalence of POP and urinary incontinence in women affected either by MS or Ehlers-Danlos Syndrome. In a series of twelve women, 4 (33%) had a history of descesus despite the young mean age (49) and the low median parity (0.5).

An observational report of Jabs and Child (2016) investigated the prevalence of POP in cohort of women with Marfan syndrome. A group of 25 women with a mean age of 43 years (range 22-59) were included in the study and 14 of them underwent also clinical examination. A total of 11 women presented POP or reported a history of descensus. One patient underwent previous Manchester repair. Another woman presented POP related symptoms but she couldn’t undergo examination. Nine patients presented POP Q stage II prolapse on physical evaluation (64% of the total physically evaluated). Six women had stage 2 anterior prolapse, which represented a recurrence in the half of cases (3) who underwent prior anterior vaginal repair for the same reason. Three patients had stage 2 posterior prolapse of whom 2 were relapses after prior vaginal prolapse repair. Follow up at 6 and 12 months showed a good support of the vaginal apex. All the patients described in this series with a history of POP reconstructive surgery presented a recurrence, reiterating the need for an extremely complex reconstructive surgery in case of MS.

This is the first case of LSHP in a woman affected by MS and the only one to describe a reconstructive procedure in this category of patients. This patient was young with an active sexual life and desire of future pregnancies. This led us to the choice of performing a conservative surgery on the uterus, to preserve the patients reproductive desire without affecting her quality of life.

Hysteropexy has been described to be a valid alternative in patients with uterine prolapse (Rahmanou et al., 2014; Campagna et al.,2021), and its safety was proved for future pregnancies (Jefferis et al., 2017). Many different surgical methods have been described in literature. In the technique described by Rahmanou et al. (2014) to correct apical prolapse, only the cervix is suspended to the sacrum, whilst in our group’s previously published technique the mesh is anchored to anterior and posterior vaginal wall (Campagna et al., 2021). In this particular case we decided to suspend the anterior and posterior vaginal wall because of the presence of a high grade multicompartimental prolapse, to better correct the anterior vaginal wall defect. Furthermore, we positioned a posterior mesh, since it is known to prevent secondary enterocele (Ercoli et al., 2016).

As proved by numerous studies, suture type used for vaginal graft attachment did not influence mesh or permanent suture exposure rates (Reisenauer et al., 2021). The high risk of relapses related to the weakness of connective tissue led us to the choice of resorting to the use of prosthetic materials and non-absorbable sutures during this laparoscopic approach, since in our experience they are associated to low complication rate and good long term outcome, avoiding surgical techniques with native tissues.

As we already specified in Materials and Methods, we asked the patient to perform a defecography to detect any rectal prolapse or intussusception, that can be found concomitantly in 10–55% of patients with POP. Frequently these problems are treated separately, leading to suboptimal outcomes. In recent years, several authors have underlined that a multidisciplinary approach to POP may improve surgical outcomes and patient symptoms, and suggested that sacrocolpopexy associated with ventral rectopexy is an effective combination of procedures for multicompartment POP. If the defecography had shown any internal rectal prolapse or rectal intussusception, we would have considered adding a rectopexy to the procedure (Campagna et al., 2020).

Hydronephrosis should also always be looked for pre-operatively, since it might be linked to ureteral kinking associated with advanced stage POP that, as described in literature, can exist up to 30% (Siddique et al., 2020) During the procedure we paid particular attention to the haemostasis avoiding hazardous or useless surgical maneuvers because the increased risk of blood loss and haematoma formation produced by the fragile vasculature (Søborg et al., 2017; Bridges et al., 1993). Urogynaecologists should take in account that LSHP is already a challenging technique because of need of deep pelvic dissections. Furthermore, the procedure might be technically more challenging due to the laxity of the tissues, making dissection harder.

In addition, abnormal fibrillin production may cause a delayed wound healing, and a higher risk for incisional hernia and dehiscence (Harrison et al., 2016).

We suggest managing these rare cases only in a tertiary referral center with high experienced surgeons. After the surgical procedure, these patients should be warned to minimise additional risk factors such as heavy lifting, constipation, weight gain or weight loss. Pelvic floor exercises should be recommended. Our case report may represent the basis of future studies to confirm the safety, efficacy and feasibility of LSHP and sacral colpopexy in patients with MS.
